# The phosphatidylserine receptor has essential functions during embryogenesis but not in apoptotic cell removal

**DOI:** 10.1186/jbiol10

**Published:** 2004-08-23

**Authors:** Jens Böse, Achim D Gruber, Laura Helming, Stefanie Schiebe, Ivonne Wegener, Martin Hafner, Marianne Beales, Frank Köntgen, Andreas Lengeling

**Affiliations:** 1 Junior Research Group Infection Genetics, German Research Center for Biotechnology (GBF), Mascheroder Weg 1, 38124 Braunschweig, Germany; 2 Department of Pathology, School of Veterinary Medicine Hannover, Bünteweg 17, 30559 Hannover, Germany; 3 Department of Experimental Immunology, German Research Center for Biotechnology (GBF), Mascheroder Weg 1, 38124 Braunschweig, Germany; 4 Ozgene Pty. Ltd., Canning Vale, WA 6970, Australia

## Abstract

**Background:**

Phagocytosis of apoptotic cells is fundamental to animal development, immune function and cellular homeostasis. The phosphatidylserine receptor (Ptdsr) on phagocytes has been implicated in the recognition and engulfment of apoptotic cells and in anti-inflammatory signaling. To determine the biological function of the phosphatidylserine receptor *in vivo*, we inactivated the *Ptdsr *gene in the mouse.

**Results:**

Ablation of *Ptdsr *function in mice causes perinatal lethality, growth retardation and a delay in terminal differentiation of the kidney, intestine, liver and lungs during embryogenesis. Moreover, eye development can be severely disturbed, ranging from defects in retinal differentiation to complete unilateral or bilateral absence of eyes. *Ptdsr *^-/-^ mice with anophthalmia develop novel lesions, with induction of ectopic retinal-pigmented epithelium in nasal cavities. A comprehensive investigation of apoptotic cell clearance *in vivo *and *in vitro *demonstrated that engulfment of apoptotic cells was normal in *Ptdsr *knockout mice, but *Ptdsr*-deficient macrophages were impaired in pro- and anti-inflammatory cytokine signaling after stimulation with apoptotic cells or with lipopolysaccharide.

**Conclusion:**

*Ptdsr *is essential for the development and differentiation of multiple organs during embryogenesis but not for apoptotic cell removal. *Ptdsr *may thus have a novel, unexpected developmental function as an important differentiation-promoting gene. Moreover, Ptdsr is not required for apoptotic cell clearance by macrophages but seems to be necessary for the regulation of macrophage cytokine responses. These results clearly contradict the current view that the phosphatidylserine receptor primarily functions in apoptotic cell clearance.

## Background

Programmed cell death, or apoptosis, is required for the normal development of almost all multicellular organisms and is a physiological mechanism for controlling cell number; as a result, structures that are no longer needed are deleted during development and abnormal cells are eliminated [1,2]. Most of the cells produced during mammalian embryonic development undergo physiological cell death before the end of the perinatal period [3]. Apoptotic cells are removed rapidly and efficiently as intact cells or apoptotic bodies by professional phagocytes or by neighboring cells. This highly regulated process prevents the release of potentially noxious or immunogenic intracellular materials and constitutes the fate of most dying cells throughout the lifespan of an organism [4,5]. Phagocytosis of apoptotic cells is very distinct from other engulfment processes that result, for example, in the clearance of microorganisms, because engulfment of apoptotic cells triggers the secretion of potent anti-inflammatory and immunosuppressive mediators, whereas pathogen recognition causes the release of pro-inflammatory signals [6].

Almost all cell types can recognize, respond to, and ingest apoptotic cells by using specific sets of phagocytic receptors that bind to specific ligands on apoptotic cells. Detailed genetic studies in *Drosophila *and *Caenorhabditis elegans *have recently yielded evidence that basic phagocytic mechanisms and pathways for the recognition and engulfment of apoptotic cells are highly conserved throughout phylogeny [7,8]. In vertebrates, a number of receptors have been identified that can mediate phagocytosis of apoptotic cells. These include, for example, scavenger receptors and pattern recognition receptors such as CD36, SR-A and CD14, integrins such as the vitronectin receptor α_v_β_3_, and members of the collectin family and their receptors CD91 and calreticulin [9-13]. The individual roles of these molecules in binding, phagocytosis or transduction of anti-inflammatory signals upon apoptotic cell recognition have not been well defined, however [5,6,14]. The importance of efficient mechanisms for apoptotic cell clearance *in vivo *is supported by the observation that autoimmune responses can be provoked in mice when key molecules for apoptotic cell recognition and uptake are missing. This has been reported for knockout mice lacking the complement protein C1q [15], for mice with a mutation in the tyrosine kinase receptor gene *Mer *[16] and, more recently, in mice lacking transglutaminase 2 or milk fat globule epidermal growth factor 8 (MFG-E8) [17,18].

The exposure of the phospholipid phosphatidylserine (PS) in the outer leaflet of the plasma membrane of apoptotic cells has been described as one of the hallmarks of the induction of apoptosis and is considered to be one of the most important signals required for apoptotic cell recognition and removal [19]. A number of cell-surface and bridging molecules can interact with exposed PS on apoptotic cells. These include the serum proteins β2-glycoprotein 1 and protein S [20,21], the growth-arrest-specific gene product GAS-6 [22], complement activation products [23], the milk fat globule protein MFG-E8 [24], and annexin I [25]. In most cases the receptors on phagocytes that recognize these PS-bridging molecules have not been defined, but it has been reported that GAS-6 is a ligand for the tyrosine kinase receptor Mer and that MFG-E8 can bind to the vitronectin receptor α_v_β_3 _[16,24]. Other molecules that bind PS with varying specificity are the lectin-like oxidized low-density lipoprotein receptor-1 (LOX-1) and the scavenger receptors CD36 and CD68 (for review see [5] and references therein).

The best-characterized molecule so far that binds PS in a stereo-specific manner is the phosphatidylserine receptor (Ptdsr) [26]. *In vitro*, it has been shown that the Ptdsr can mediate the uptake of apoptotic cells and that such Ptdsr-mediated phagocytosis can be inhibited through addition of PS liposomes, the PS-binding molecule annexin V or an anti-Ptdsr antibody [26]. Moreover, the binding of Ptdsr to PS on apoptotic cells has been reported to be important for the release of anti-inflammatory mediators, including transforming growth factor-β1 (TGF-β1), platelet-activating factor (PAF), and prostaglandin E2 [26,27]. These data supported the hypothesis that Ptdsr fulfils a role as a crucial signaling switch after the engagement of macrophages with apoptotic cells and is thereby fundamental for preventing local immune responses to apoptotic cells before their clearance [28].

Very recently, Ptdsr has been found in the cell nucleus. Its nuclear localization is mediated by five independent nuclear localization signals, each of which alone is capable of targeting Ptdsr to the cell nucleus [29]. Moreover, an additional study performed recently in *Hydra *showed an exclusively nuclear localization for the Ptdsr protein [30]. Most interestingly, the nuclear localization of Ptdsr in *Hydra *epithelial cells did not change upon phagocytosis of apoptotic cells. These reports challenge the original hypothesis, according to which Ptdsr is an exclusively transmembrane receptor for apoptotic cell recognition and anti-inflammatory signaling.

To examine further the role of Ptdsr *in vivo*, we performed gene-expression and gene-targeting studies in mice. A perinatally lethal phenotype was observed in *Ptdsr-*knockout mice, and *Ptdsr*-deficient embryos displayed multiple defects in tissue and organ differentiation. While this work was in progress, both Li *et al. *[31] and Kunisaki *et al. *[32] also reported the generation and phenotypic characterization of *Ptdsr*-knockout mice. Of note, although some of their results were confirmed in our study, we found a fundamentally different phenotype with regard to clearance of apoptotic cells. Moreover, our study revealed marked and unexpected findings in *Ptdsr*-deficient mice that are not related to apoptosis.

## Results

### Generation of *Ptdsr*-deficient mice

**Figure 1:**
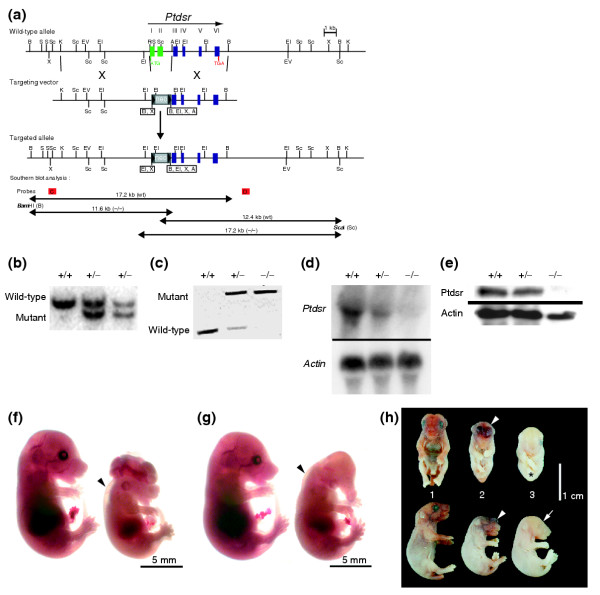
Targeted inactivation of the phosphatidylserine receptor gene. **(a) ***Ptdsr *gene-targeting strategy. Homologous recombination in ES cells results in the deletion of exons I and II of the murine *Ptdsr *gene through replacement of a *loxP*-flanked neomycin phosphotransferase gene (neo), thereby ablating the reading frame of the encoded protein. Coding exons I-VI are shown as filled boxes, and deleted exons are colored green. Restriction sites are: A, *Aat*II; B, *Bam*HI; EI, *Eco*RI; EV, *Eco*RV; K, *Kpn*I; R, *Rsr*II; S, *Sac*II; Sc, *Sca*I, X, *Xho*I. The probe sites are red boxes labeled: C, 5' outside probe; D, 3' outside probe. **(b) **Southern blot analysis of genomic DNA extracted from wild-type (+/+) and *Ptdsr*^+/- ^(+/-) animals, digested with *Bam*HI and hybridized with the 5' outside probe to confirm germ-line transmission of the mutant *Ptdsr *allele. 'Wild-type' indicates the *Bam*HI fragment of 17.2 kb from the wild-type *Ptdsr *allele; 'mutant' indicates the *Bam*HI fragment of 11.6 kb from the targeted *Ptdsr *allele. **(c) **PCR genotyping of embryos and animals from intercrosses of heterozygous *Ptdsr*^+/- ^using a wild-type and a mutant allele-specific primer combination, respectively. **(d) **Northern blot analysis of total RNA isolated from E13.5 wild-type, *Ptdsr*^+/- ^and *Ptdsr *^-/- ^embryos. **(e) **Western blot analysis of protein from homogenates of E13.5 wild-type, *Ptdsr*^+/- ^and *Ptdsr *^-/- ^embryos using a Ptdsr-specific antibody. Developmental abnormalities at **(f,g) **E15.5 and **(h) **birth; in this and all subsequent figures wild-type littermates are located on the left and homozygous mutant mice on the right. The *Ptdsr *^-/- ^ embryos show exencephaly (f) or prosencephalic hernia in the forebrain region (arrowhead, neonate 2; h), uni-or bilateral absence of the eyes (f,g and neonate 2 in h, and arrow, neonate 3 in h), an abnormal head shape with proboscis (g), edema (arrowheads in f and g), and general anemia (asterisk, neonate 3 in h).

To investigate *in vivo *the functions of the phosphatidylserine receptor Ptdsr, we generated a null allele in the mouse by gene targeting (Figure 1a,1b,1c). In contrast to previously described *Ptdsr-*knockout mice [31,32], we used Bruce4 embryonic stem (ES) cells for gene targeting [33], thus generating a *Ptdsr*-null allele in a pure, isogenic C57BL/6J genetic background. The newly established knockout mouse line was named *Ptdsr*^*tm1Gbf *^(hereafter referred to as *Ptdsr *^-/-^). Heterozygous *Ptdsr*^+/- ^mice were viable and fertile and showed no obvious abnormalities. *Ptdsr *^+/- ^mice were intercrossed to generate homozygous *Ptdsr*-deficient mice. The absence of *Ptdsr *expression in *Ptdsr *^-/- ^embryos was confirmed by RT-PCR (data not shown), and by northern and western blotting analyses (Figure 1d,e). Interbreeding of heterozygous mice showed that the mutation was lethal, since homozygous mutants were not detected in over 100 analyzed litters at weaning. To determine the stages of embryonic development affected by the *Ptdsr*^*tm1Gbf *^mutation, timed breedings were followed by PCR genotyping (Figure 1c) of embryos. We recovered fewer than the expected number of homozygous embryos from intercrosses of *Ptdsr*^+/-^mice. From a total of 1,031 embryos analyzed between gestational day (E) 9.5 and E18.5, 198 (19.2%) *Ptdsr*-deficient homozygous embryos were harvested, indicating that the introduced mutation is associated with a low rate of embryonic lethality *in utero.*

From E9.5 to E12.5, *Ptdsr *^-/- ^ embryos were viable and of normal size. At E13.5 and thereafter, however, most *Ptdsr *^-/-^embryos showed morphological abnormalities (Table 1). All homozygous embryos harvested were growth-retarded from E13.5 onwards, had a pale appearance, and displayed multiple developmental dysmorphologies. These included various head and craniofacial malformations, such as exencephaly, cleft palate and abnormal head shape (Figure 1f,g). Gross inspection revealed that eye development was severely affected in 14.1% of homozygous embryos. The affected animals displayed a complete unilateral or bilateral absence of the eyes (Table 1) that was never detected in *Ptdsr *^+/+ ^or *Ptdsr *^+/- ^littermates. Furthermore, homozygous embryos harvested between E12.5 and E15.5 had subcutaneous edema (Figure 1f,g). Because we were able to recover *Ptdsr *^-/-^ embryos until E18.5, we investigated whether *Ptdsr*-knockout mice could be born alive. Careful observation of timed matings allowed us to recover *Ptdsr *^-/- ^neonates, but homozygous pups died during delivery or within minutes after birth. *Ptdsr*-deficient neonates were also growth-retarded, had a pale appearance and displayed various malformations. These included cleft palate, abnormal head shape, absence of eyes and edematous skin (Figure 1h). Thus, deletion of the *Ptdsr *gene resulted in perinatal lethality with variable severity and penetrance of phenotypes.

### Expression of *Ptdsr *during embryogenesis and in adult tissues

**Figure 2:**
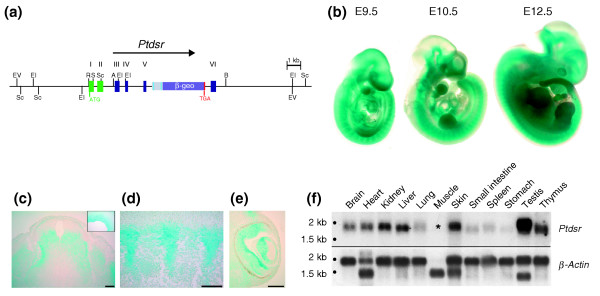
Expression analysis of *Ptdsr *during embryonic development. **(a**) Schematic representation of the construction of the *Ptdsr *gene-trap mouse line used for expression analysis at different embryonic stages. Gray and bright blue boxes represent regulatory elements of the gene-trap, and β-geo, the β-galactosidase/neomycin phosphotransferase fusion protein-expression cassette [48,51]. Restriction enzyme nomenclature is as in Figure 1 **(b) **Whole-mount β-galactosidase staining of heterozygous *Ptdsr *gene-trap embryos at mid-gestation. Expression of *Ptdsr *is highest in neural tissues and somites, in the branchial arches, the developing limbs, the heart, the primitive gut and the developing eye. **(c-e) **Sectioning of E12.5 β-galactosidase-stained embryos confirms expression of *Ptdsr *in (c) the neural tube; (inset in c) neural epithelium; (d) somites; and (e) eyes. Expression in the eye is restricted to developing neural retinal and lens cells. **(f) **Expression analysis of adult tissues by northern blot. Expression of *Ptdsr *in the muscle (asterisk) was detected only on long-term exposures of the filter (> 48 h). A *β-actin *hybridization was used to confirm equal loading of RNA samples. Scale bar, 100 μm.

The observed perinatal lethality indicates that *Ptdsr *plays an important role during development. Analysis by RT-PCR (data not shown) showed that *Ptdsr *is expressed early in development, because we were able to detect *Ptdsr *transcripts in ES cells and embryos at all developmental stages. To analyze in more detail the temporal and spatial expression patterns of *Ptdsr*, and to correlate expression patterns with observed pathological malformations, we made use of a *Ptdsr-β-geo *gene-trap reporter mouse line generated from a *Ptdsr *gene-trap ES cell clone. This line has an insertion of *β-galactosidase *in the 3' region of the gene (Figure 2a).

We first examined *Ptdsr *expression by X-Gal staining in heterozygous embryos staged from E9.5 to E12.5. These developmental stages were chosen so as to investigate *Ptdsr *expression in affected organs prior to the onset of pathological malformations in *Ptdsr *^-/- ^embryos. At E9.5 we found *Ptdsr *expression in the developing neural tube, somites, heart, gut and branchial arches (Figure 2b). At E10.5, *Ptdsr *expression remained high in the developing nervous system, with most intense staining in the forebrain, hindbrain and neural tube. At this stage of embryogenesis, high levels of *Ptdsr *expression could also be detected in the developing limb buds and eyes (Figure 2b). *Ptdsr *expression was altered at E12.5, with most intensive β-galactosidase staining in the eyes, developing condensations of the limb buds, neural tube and brain (Figure 2b). Transverse sections of X-Gal-stained embryos at E12.5 showed an asymmetric expression pattern in the neural tube with intense staining of the central mantle layer but no expression in the dorsal part of the neural tube (for example, the roof plate; Figure 2c). Expression in dorsal root ganglia lateral to the neural tube and in the somites was observed; *Ptdsr *was expressed throughout the somite structure (myotome, dermatome and sclerotome; Figure 2d). Expression boundaries between somites were evident, with no expression in the segmental interzones, which correspond to the prospective intervertebral discs (Figure 2d). Transverse sections of the developing eye at E12.5 revealed strong *Ptdsr *expression in the inner layer of the neural cup, which will later develop into the neural retina. Furthermore, *Ptdsr *expression was detected in the primary lens fiber cells of the developing lens (Figure 2e). We carefully investigated whether *Ptdsr *is expressed from E10.5 to E12.5 in the developing kidney and lungs, but no expression could be detected indicating that *Ptdsr *expression is required only at later stages in the development of these organs (see below).

Hybridization of a multiple-tissue northern blot revealed a single transcript of about 1.8 kb in almost every tissue analyzed in adult mice (Figure 2f). The most prominent expression was observed in testis, thymus, kidney, liver and skin, with moderate to low expression in lung, small intestine, spleen, stomach and skeletal muscle. Thus, *Ptdsr *is ubiquitously expressed throughout embryogenesis and in adult tissues, although at different levels.

### *Ptdsr *is required for normal tissue and organ differentiation

**Figure 3:**
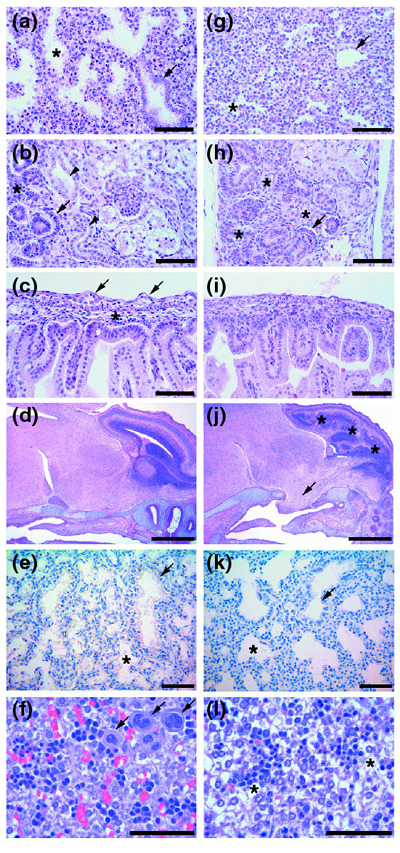
Histological analysis of wild-type and *Ptdsr *^-/-^organs during embryogenesis. **(a-f) **Wild-type embryos and **(g-l) ***Ptdsr *^-/- ^littermates were isolated at various embryonic stages, serially sectioned sagittally and analyzed for developmental abnormalities in detail after H&E staining. At E16.5, the lungs of (g) *Ptdsr *^-/- ^embryos had sacculation just starting, and well-formed alveoli (asterisks) or epithelium-lined bronchioles (arrows) were scarce compared to (a) wild-type lungs. At E16.5, the glomeruli (arrows) in the kidney of (h) *Ptdsr *^-/- ^embryos were underdeveloped compared to (b) wild-type, collecting tubules (arrowheads) were missing and undifferentiated blastemas (asterisks) were more abundant. The jejunum had no intramural ganglia in *Ptdsr *^-/-^embryos (i; and arrows in c); and a well-developed submucosa (asterisk in c) was missing. Brain sections at E18.5 show that (j) *Ptdsr *^-/-^embryos may have herniation (arrow) of the hypothalamus through the ventral skull (secondary palate), most likely through Rathke's pouch, and a severe malformation of the cortex (asterisks) compared to (d) wild-type embryos. At E18.5, (e) wild-type and (k) *Ptdsr *^-/- ^lungs showed normal sacculation and formation of alveoli (asterisks) and bronchioles (arrow). (f) Wild-type neonatal liver had significant numbers of megakaryocytes (arrows), compared to (l) homozygous mutant littermates, and higher numbers of erythropoietic islands and of mature erythrocytes. Hepatocellular vacuoles are due to glycogen stores (asterisks) that were not metabolized in perinatally dying *Ptdsr *^-/- ^animals, in contrast to wild-type newborns. Scale bar, 100 μm, except for (d) and (j), 1 mm.

We next examined the role of *Ptdsr *in organ development. Serial histological sections of *Ptdsr *^-/- ^and control embryos were taken to perform a detailed morphological analysis of all organ systems during development. A significant delay in organ and tissue differentiation was observed at E16.5 in lungs, kidneys and intestine. Lungs of control littermates were properly developed with expanding alveoli (Figure 3a). Terminal bronchi and bronchioles were already well developed, and terminally differentiated epithelial cells with cilia on the luminal cell surface were present. In contrast, almost no alveoli or bronchioles were present in *Ptdsr *^-/- ^ lungs, indicating a delay or arrest in lung sacculation and expansion. Instead, we observed an abundance of mesenchyme that appeared highly immature (Figure 3g). A similar delay in tissue differentiation of *Ptdsr *^-/- ^embryos was found in the kidneys (Figure 3h). Kidneys from *Ptdsr*^+/+ ^ embryos were well developed at E16.5, showing terminally differentiated glomeruli with Bowman's capsule and collecting tubules lined with cuboidal epithelial cells (Figure 3b). In contrast, *Ptdsr*-deficient kidneys had only primitive glomeruli at E16.5, and collecting tubules were less well-developed. Instead, a large amount of undifferentiated mesenchyme was present in *Ptdsr *^-/- ^kidneys (Figure 3h). A delay in tissue differentiation was also found in the intestine at this stage of development. *Ptdsr *^-/- ^embryos displayed improperly developed villi and an underdeveloped or absent submucosa (Figure 3i). In wild-type embryos (Figure 3c), intestinal cellular differentiation was already highly organized, with intramural ganglion cells between the external and internal muscular layers. Such neuronal cells were absent from the intestine of *Ptdsr *^-/- ^embryos (Figure 3i), however.

Some *Ptdsr *^-/- ^mice (4.5 %) also displayed extensive brain malformations that resulted in externally visible head abnormalities, with occasional ectopic tissue outside the skull or exencephaly (Figure 1f,h). Histological analysis revealed an extensive hyperplasia of brain tissue with herniation of brain tissue either through the skull-cap or through the ventral skull (Figure 3d,j). In the most severe cases, expansion of brain tissue in mutant mice resulted in further perturbations of cortical structures (Figure 3d,j). Of note, a similar brain phenotype was observed in the *Ptdsr*-deficient mouse line generated by Li and colleagues [31].

In contrast to the study of Li *et al. *[31], however, we found almost normally developed lungs at birth. *Ptdsr *^-/-^lungs showed, in comparison to wild-type, only a slight delay in maturation and were fully ventilated in neonates in most cases (Figure 3e,k). This demonstrates that *Ptdsr*-deficient mice can overcome the delay in embryonic lung differentiation and display normal lung morphology at birth. Thus, it would appear highly unlikely that *Ptdsr *^-/-^ mice die from respiratory failure. Consistent with the observations of Kunisaki and colleagues [32], we found severely blocked erythropoietic differentiation at an early erythroblast stage in the liver (Figure 3f,3l), suggesting an explanation for the grossly anemic appearance that we observed in our *Ptdsr *^-/- ^mice.

### Loss of *Ptdsr *activity is associated with defects in ocular development and can lead to formation of ectopic eye structures

**Figure 4:**
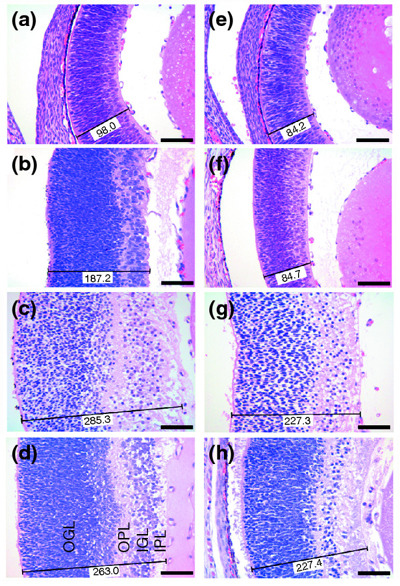
Morphology of wild-type and *Ptdsr *^-/- ^retinas. Serial sagittal sections of **(a-d) **wild-type and **(e-h) ***Ptdsr *^-/- ^retina were analyzed for developmental abnormalities at (a,e) E12.5, (b,f) E16.5, (c,g) E18.5, and (d,h) P0. Normal patterning of the retina was observed in *Ptdsr *^-/-^embryos, with an outer granular layer (OGL), outer plexiform layer (OPL), inner granular layer (IGL) and inner plexiform layer (IPL). Note that the IGL in *Ptdsr *^-/- ^retinas is less thick than that in wild-type littermates in comparing (c,g) and (d,h). Morphometric analysis (numbered lines) of wild-type and *Ptdsr *^-/- ^retinas confirmed the initial finding of a thinner retina in *Ptdsr *^-/- ^animals than in wild-type (all values in μm). Scale bar, 50 μm.

**Figure 5:**
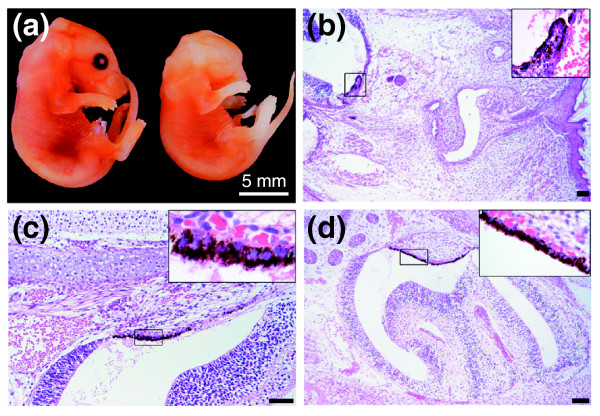
Histological analysis of eye development in severely affected eyeless *Ptdsr *^-/- ^embryos. **(a) **In anophthalmic *Ptdsr *^-/- ^embryos, unilateral or bilateral absence of the eyes could be detected. **(b-d) **Serial H&E-stained sagittal sections of homozygous mutant embryos at (b) E17.5 and (c,d) E18.5 show complex malformation of the optic cup and lack of any lens structure. Careful examination of adjacent sections (b-d) reveals an ectopic misplacement of retinal-pigmented epithelium in the maxillary sinus. Not only is the deposition of pigment clearly visible (higher magnification insets) but also the induction of proliferation of underlying tissues and the change in morphology of the maxillary sinus (d). Scale bar, 100 μm in (b-d).

By gross morphology we could differentiate two classes of *Ptdsr *mutants: those that appeared normal with both eyes present (Figure 4) and those that were severely affected and displayed uni- or bilateral anophthalmia (Figure 5).

Analysis of normal or mildly affected embryos revealed no differences between mutant and wild-type embryos in the differentiation of the developing eye until E16.5. In both genotypes, inner and outer layers of the retina displayed a comparable differentiation status, as shown, for example, at E12.5 (Figure 4a,e). At day E16.5, however, retinal layers in *Ptdsr *^-/- ^embryos were much thinner than in wild-type embryos, contained fewer cells and were greatly reduced in size (Figure 4b,f). Comparison of the retinal structures of *Ptdsr *^+/+ ^and *Ptdsr *^-/- ^embryos revealed that all four retinal layers were present in *Ptdsr*-knockout mice at E16.5 (Figure 4b,f). At E18.5 (Figure 4c,g) and in neonatal animals (postnatal day P0; Figure 4d,h), the differences in retinal differentiation between *Ptdsr*^+/+ ^and *Ptdsr *^-/- ^mice were still evident, but the size reduction of the retinal layers was less pronounced in the knockout mice. *Ptdsr*-deficient animals seem to have compensated for the marked delay in cellular differentiation and expansion of retinal layers. Close examination of retinal structures revealed that the inner granular layer was still less expanded in *Ptdsr*-deficient animals, however, and that it contained fewer cells and was still severely underdeveloped in comparison with the corresponding retinal layer in control animals (Figure 4c,4g and 4d,4h). Thus, even mildly affected *Ptdsr *^-/- ^mutants had ocular malformations with defects in differentiation of retinal structures.

We next examined *Ptdsr *^-/- ^embryos that displayed unilateral or bilateral absence of eyes (Figure 5a) by serial sectioning of whole embryos. These embryos showed complex malformations of the optical cup, including absence of the lens (Figure 5b). Most surprisingly, we found pigmented epithelial cells in the nasal cavity of all *Ptdsr*-knockout mice with anophthalmia that were analyzed histopathologically. We could identify black-colored pigmented cells embedded in the epithelium of the maxillary sinus that resembled presumptive retinal-pigmented epithelium (Figure 5b,c). Examination of consecutive serial sections revealed the formation of a primitive eye structure, with induction and subsequent proliferation of ectopic mesenchymal tissue immediately adjacent to the displaced pigmented epithelium (Figure 5d). This structure was clearly induced ectopically, and we failed to identify similar changes in any of the wild-type embryos. In summary, we observed a wide range of ocular malformations in *Ptdsr*-deficient mice that ranged from differentiation defects in retinal cell layers (for example, the inner granular layer) in mildly affected homozygotes to anophthalmia in severely affected *Ptdsr *^-/- ^mice that was associated with induction of ectopic eye structures in nasal cavities.

### Phagocytosis and clearance of apoptotic cells is normal in *Ptdsr*-deficient mice

**Figure 6:**
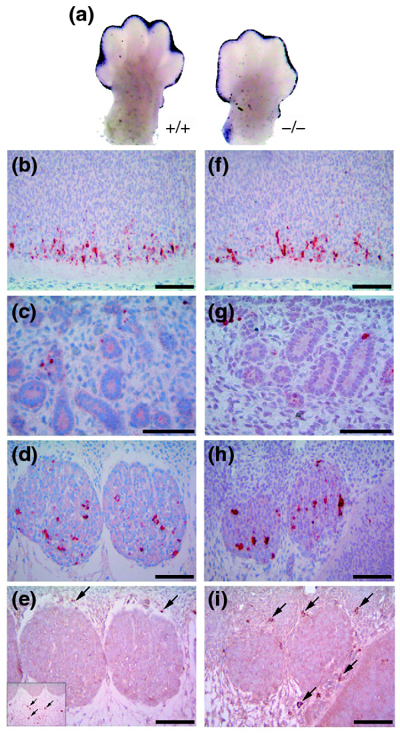
Analysis of programmed cell death and involvement of macrophages in the removal of apoptotic cells in wild-type and *Ptdsr *^-/-^embryos. **(a) **Whole-mount TUNEL staining (blue) of limb buds from wild-type and *Ptdsr *^-/- ^embryos at E13.5 show no differences in the amount or localization of apoptotic cells during the beginning regression of the interdigital web. Serial sagittal sections stained for activated caspase 3 (aCasp3; red) in **(b-d) **wild-type and **(f-h) ***Ptdsr *^-/- ^embryos at E12.5 show apoptotic cells in the neural tube (b,f), the mesonephros (c,g) and the developing paravertebral ganglia (d,h). Tissue distribution and total number of apoptotic cells was indistinguishable between genotypes and was confirmed by the comparison of consecutive sections of wild-type and *Ptdsr *^-/-^embryos from different developmental stages. Analysis of macrophage numbers and location by F4/80 staining (brown) of consecutive sections in paravertebral ganglia of **(e)** wild-type and **(i)** homozygous mutant embryos revealed that macrophages (arrows) are not located close to apoptotic cells during embryonic development. (For comparison, see also Additional data file 1, Figure S1, with the online version of this article). Scale bar, 100 μm.

We next tested whether *Ptdsr *is functionally required for the clearance of apoptotic cells. We started with an investigation of cell death *in vivo *in the interdigital areas of the developing limbs. Apoptosis of interdigital cells in the distal mesenchyme of limb buds occurs most prominently from developmental stages E12.0 to E13.5 and can be easily examined *in situ *by whole-mount terminal deoxynucleotide transferase-mediated UTP end-labeling (TUNEL). We compared the pattern of interdigital cell death in fore and hind limb buds from *Ptdsr *^-/-^ (*n *= 3) and *Ptdsr *^+/+ ^(*n *= 3) mice at E12.5 and E13.5. No differences in accumulation of TUNEL-positive cell corpses were observed between the two genotypes (Figure 6a). The kinetics of cell death occurrence and regression of the interdigital web was similar in wild-type and mutant littermates, providing no evidence that *Ptdsr*-deficiency is associated with impaired clearance of apoptotic interdigital cells during limb development.

To investigate further whether removal of apoptotic cells is impaired in *Ptdsr *^-/- ^mice, we stained immunohistochemically for activated caspase 3 (aCasp3) and analyzed additional organs and tissues where apoptosis plays a crucial role in tissue remodeling during development. Starting at E12.5, we analyzed and compared the number and distribution of aCasp3-positive cells in over 140 serial sections of three wild-type and six *Ptdsr *^-/- ^embryos in consecutive and corresponding sections. The sagittal sections were separated by 5 μm, allowing a detailed analysis of apoptosis in several organs and tissues. Tissue restructuring by programmed cell death occurred most notably within the ventral part of the neural tube (Figure 6b,f) and in the developing paravertebral ganglia (Figure 6d,h) with many apoptotic cells being present. In these tissues Ptdsr is highly expressed at E12.5 (Figure 2c) but we observed no difference in the number or distribution of apoptotic cells in *Ptdsr*^+/+ ^and *Ptdsr *^-/- ^embryos. The same was true for the developing kidney: apoptotic cells were present in *Ptdsr*^+/+ ^and *Ptdsr *^-/- ^embryos, in limited numbers, but we failed to detect any differences in the number of apoptotic cells between the genotypes (Figure 6c,6g). Furthermore, when we continued our analysis of apoptotic cell clearance *in vivo *at E16.5, E17.5 and E18.5 of embryonic development as well as in neonatal mice, the number and distribution of apoptotic cells was similar in both genotypes. As already observed at E12.5, analysis of aCasp3-stained sections of the developing thymus, heart, diaphragm, genital ridge, eyes and retina convincingly showed that there was no impairment in apoptotic cell removal in *Ptdsr *^-/- ^mice. Moreover, because Li and colleagues [31] reported impaired clearance of dead cells during lung development in *Ptdsr*-deficient mice, we examined the rate of apoptosis induction and cell clearance in our *Ptdsr*-knockout mice in the lung. Analysis of aCasp3-stained lung tissue from *Ptdsr*^+/+ ^and *Ptdsr *^-/- ^mice at E17.5 and P0 demonstrated that apoptosis was an extremely rare event during lung morphogenesis at this stage. In addition, there were no differences in the number or distribution of apoptotic cells in *Ptdsr *^-/- ^and *Ptdsr *^+/+ ^mice. Furthermore, we were unable to detect any evidence of tissue necrosis in lungs from *Ptdsr*-deficient mice. In contrast to the report of Li *et al. *[31], we never observed recruitment of neutrophils or other signs of pulmonary inflammation at any stage of development in our *Ptdsr*-deficient mice.

To analyze whether macrophages are recruited into areas where apoptosis is prominent during embryogenesis, we stained consecutive serial sections either with the macrophage surface marker F4/80 or with aCasp3. Surprisingly, there was no co-localization of macrophages with apoptotic cells. In virtually all embryonic tissues, apoptotic cells and macrophages were localized in different compartments (Figure 6e,6i; and see also Additional data file 1, Figure S1). This suggests that at this stage of development it is mainly neighboring cells that are involved in removal of apoptotic cells, rather than professional macrophages. In summary, our analysis *in vivo *did not reveal any impairment in apoptotic cell clearance in *Ptdsr*-deficient embryos during development and further suggests that phagocytosis of apoptotic cells is mainly mediated by non-professional 'bystander' cells.

**Figure 7:**
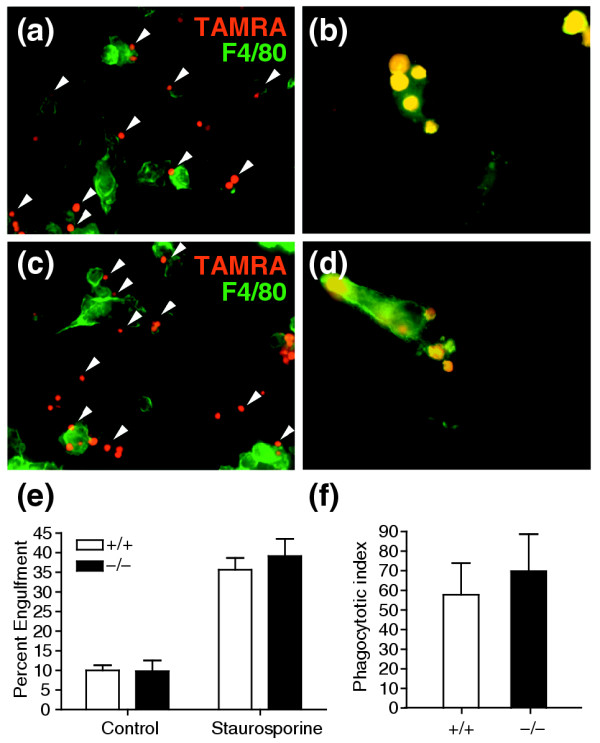
Phagocytosis of apoptotic cells by fetal liver-derived macrophages (FLDMs). FLDMs from **(a,b) **wild-type and **(c,d) ***Ptdsr *^-/- ^embryos were cultured for 60 min with TAMRA-stained (red) apoptotic thymocytes (treated with staurosporine) from C57BL/6J mice and then stained with F4/80 (green). Macrophages of both genotypes have phagocytosed apoptotic cells (arrowheads). **(e) **Quantification of phagocytosis of apoptotic cells by wild-type or *Ptdsr *^-/-^macrophages revealed no differences in the percentage of macrophages that had engulfed apoptotic cells, whether or not apoptosis had been induced by staurosporine. Microscopic analysis (b,d) and quantification of the number of apoptotic cells phagocytosed by single macrophages and **(f) **calculation of the average number of cells phagocytosed per macrophage failed to reveal differences in the efficacy of removal of apoptotic cells between wild-type and *Ptdsr *^-/- ^ FLDMs.

To determine whether macrophages from *Ptdsr*-knockout mice were impaired in the efficacy of apoptotic cell uptake *in vitro*, we performed phagocytosis assays with fetal-liver-derived macrophages (FLDMs) and quantified their phagocytosis rates. Phagocytosis of apoptotic thymocytes was investigated at 60, 90 and 120 minutes after addition of target cells in the absence of serum. Analysis of phagocytosis rates by flow cytometric analysis (FACS) revealed no differences in the efficacy of apoptotic cell uptake between *Ptdsr *^-/-^ and *Ptdsr*^+/+^ macrophages and demonstrated no differences in apoptotic cell engulfment between selected time points (data not shown). To re-examine and further independently validate the result of normal apoptotic cell uptake by *Ptdsr *^-/-^ macrophages, we performed phagocytosis assays for 60 min and determined the percentage of macrophages that had engulfed apoptotic cells, in a total of at least 300 macrophages counted by fluorescence microscopy. Phagocytosed, 5-carboxytetramethylrhodamine- (TAMRA-) labeled apoptotic cells were identified as being engulfed by inclusion in F4/80-labeled macrophages. Analysis was done independently by three investigators who were not aware of macrophage genotypes (*Ptdsr *^-/- ^or *Ptdsr*^+/+^). Again, no differences were found in the percentage of macrophages that had engulfed apoptotic cells (Figure 7a,c,e) or in the relative number of phagocytosed apoptotic cells per macrophage (phagocytotic index; Figure 7f). Moreover, single *Ptdsr *^-/-^macrophages could be identified that had engulfed even more apoptotic target cells than had wild-type macrophages (Figure 7b,d). Thus, *Ptdsr*-deficient macrophages had a normal ability to ingest apoptotic cells and were not impaired in recognition or phagocytosis of cells that had undergone programmed cell death.

### *Ptdsr*-deficiency results in reduced production of pro- and anti-inflammatory cytokines after macrophage stimulation

**Figure 8:**
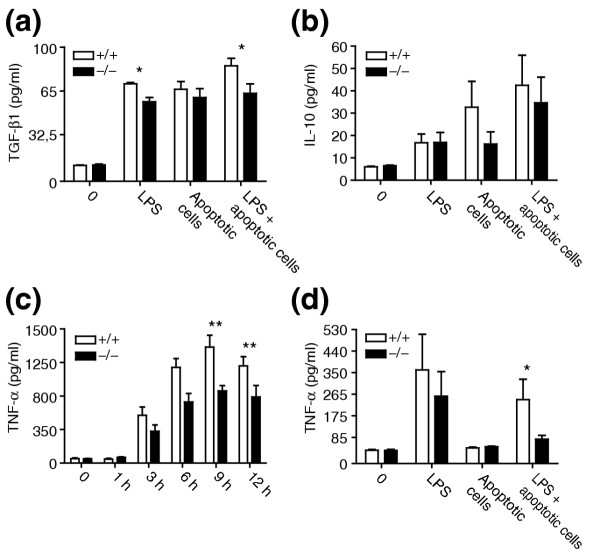
Cytokine production by FLDMs upon stimulation with lipopolysaccharide (LPS) and apoptotic cells. FLDMs from wild-type and *Ptdsr *^-/- ^embryos were incubated (a,b,d) with medium (0), LPS (10 ng/ml), apoptotic cells (ratio 1:10) or in combination with LPS and apoptotic cells or (c) with LPS (100 ng/ml) alone. Culture supernatants were harvested after 22 h (a,b,d) or at the indicated time points (c). TNF-α and TGF-β1 were quantified by ELISA and IL-10 by cytometric bead array (CBA) assay. Data are presented as mean ± SEM from at least three independent experiments, each carried out in triplicate. *, significant difference between genotypes, *p *< 0.05; **, significant difference between genotypes, *p *< 0.01; Wilcoxon-signed rank test.

In addition to its suggested importance for phagocytosis of apoptotic cells, it has been proposed that Ptdsr fulfils a second crucial role in regulating and maintaining a non-inflammatory environment upon the recognition of apoptotic cells by macrophages [26]. We therefore tested whether *Ptdsr *^-/- ^macrophages were able to release anti-inflammatory cytokines after ingestion of apoptotic cells. We examined levels of TGF-β1 and interleukin-10 (IL-10) after stimulation of FLDMs with lipopolysaccharide (LPS), with and without co-culture of apoptotic cells. Quantification of TGF-β1 and IL-10 levels after 22 hours of culture demonstrated that *Ptdsr *^-/- ^macrophages were able to secrete these anti-inflammatory cytokines upon ingestion of apoptotic cells, although at a slightly lower level than wild-type (Figure 8a,b). This indicates that ablation of *Ptdsr *function does not compromise in general the ability of macrophages to release immune-suppressive cytokines after recognition and engulfment of apoptotic cells.

To analyze whether pro-inflammatory signaling is affected in *Ptdsr *^-/- ^macrophages, we stimulated FLDMs from *Ptdsr*^+/+ ^and *Ptdsr *^-/- ^mice with LPS and measured levels of tumor necrosis factor-α (TNF-α) at different time points after stimulation (Figure 8c). *Ptdsr *^-/- ^macrophages produced significantly less TNF-α than did wild-type macrophages. The difference in TNF-α secretion was first visible after 3 h of LPS stimulation and became more prominent during the course of the experiment (for example, after 9 h and 12 h of LPS stimulation; Figure 8c). To analyze whether TNF-α release by *Ptdsr *^-/- ^macrophages can be affected by engulfment of apoptotic cells, we stimulated FLDMs with LPS, apoptotic cells or both. Quantification of TNF-α levels by ELISA after 22 h showed that *Ptdsr*-deficient macrophages release less TNF-α after stimulation with LPS alone, and also after double stimulation of macrophages with LPS and apoptotic cells (Figure 8d). Moreover, the double stimulation demonstrated that the LPS-induced TNF-α release by *Ptdsr *^-/- ^macrophages could be inhibited by co-administration of apoptotic cells to an extent comparable to that seen in wild-type macrophages. Similar results were obtained when other pro-inflammatory cytokines, such as interleukin-6 and monocyte chemoattractant protein-1, were analyzed (data not shown). These results indicate that Ptdsr is not required in macrophages for the inhibition of pro-inflammatory signaling after recognition and engulfment of apoptotic cells. *Ptdsr*-deficiency does, however, affect the overall release of pro- and anti-inflammatory cytokines after stimulation with LPS and after double treatment with LPS and apoptotic cells, indicating that *Ptdsr*-deficient macrophages have a reduced capacity to produce or secrete pro- and anti-inflammatory cytokines.

## Discussion

### *Ptdsr *is required for the differentiation of multiple organ systems during development

In this study, we have generated a null mutation in the *phosphatidylserine receptor *(*Ptdsr*) gene in C57BL/6J mice. We show that ablation of *Ptdsr *results in profound differentiation defects in multiple organs and tissues during embryogenesis, although with variable penetrance. While this work was in progress, two other groups reported the generation of *Ptdsr*-deficient mice [31,32]. In all three knockout mouse lines, the first two exons ([31] and this study) or exons one to three [32] were deleted by replacement with a neomycin-selection cassette. The *Ptdsr*-knockout mouse lines differ in the genetic background in which the mutation was generated and maintained, however. In our case, the *Ptdsr*-null allele was generated in an isogenic C57BL/6J background, whereas Li *et al. *[31] and Kunisaki *et al. *[32] investigated the phenotype of their *Ptdsr*-knockout mice in a mixed 129 × C57BL/6 background. The ablation of *Ptdsr *function results in perinatal lethality in all cases, but there are interesting differences in severity or expressivities of phenotypes among the different *Ptdsr*-deficient mouse lines. This might be due either to differences in genetic background or because the phenotypes that have been investigated in this study have not been analyzed in such detail before.

In the *Ptdsr*-knockout mouse line reported here, growth retardation started from E12.5 onwards and was associated with delayed differentiation in several organs in which *Ptdsr *is expressed either during embryogenesis or later in adulthood. At E16.5 almost no branching morphogenesis of the lung epithelium was observed in *Ptdsr *^-/-^ lungs. Similarly, epithelial structures were only partially developed in mutant kidneys, without terminal differentiation of Bowman's capsule and with a severe reduction in the number of differentiated collecting tubules. Likewise, the differentiation of the intestine was also severely delayed at this developmental stage. When compared with wild-type controls, intestinal tissues of *Ptdsr *knockout mice appeared unstructured, with an absence of enteric ganglia and of differentiated smooth muscle tissue. Interestingly, defects in kidney and intestine differentiation were not described in the *Ptdsr*-knockouts generated by Li *et al. *[31] and Kunisaki *et al. *[32]. Surprisingly, when we examined *Ptdsr*^-/- ^embryos shortly before birth (E18.5) or neonatally, we found only mild differentiation delays in organs that appeared severely affected at mid-gestation. This 'recovery' was most visible in *Ptdsr *^-/- ^lungs: at P0 we found expanded lungs in the knockout mice that showed normal branching patterns, with differentiated alveoli and bronchioles.

We investigated the occurrence of programmed cell death during lung development in wild-type and *Ptdsr*-knockout mice throughout embryogenesis (E16.5 to P0). Comparative immunohistochemistry for aCasp3 revealed that apoptosis is a rare event during lung morphogenesis. Furthermore, we failed to detect any differences in the number of apoptotic cells in *Ptdsr*-knockout and wild-type animals in the rare cases where we could detect apoptotic cells within lung tissues. These findings are contrary to the results reported by Li *et al. *[31], who suggested that impaired clearance of apoptotic mesenchymal and epithelial cells causes a failure in lung morphogenesis in *Ptdsr*-deficient mice. In contrast, our findings are in line with the current view on lung development during embryogenesis. Accordingly, formation of the epithelial lung via branching morphogenesis can be subdivided into a series of sequential steps that involve: first, formation of the organ anlage in the form of a placode; second, primary bud formation by placode invagination; third, branch initiation and branch outgrowth; fourth, further reiteration of the branching process; and fifth, terminal differentiation of organ-specific proximal and distal structures [34,35]. In contrast to other invagination processes during embryogenesis, such as mammary gland formation, the lumen of the lungs is expanded by successive branching events, branch outgrowth and elongation, rather than by apoptosis [34,36]. Finally, because the lungs of *Ptdsr *^-/-^neonates were almost fully expanded and appeared normal in structure in comparison to wild-type littermates, it is highly unlikely that *Ptdsr *mutants die of respiratory lung failure. In addition, Li and colleagues [31] demonstrated that surfactant expression is normal in *Ptdsr*-deficient animals, supporting the idea of normal maturation of surfactant-producing type II alveolar epithelial cells and lung function. Other defects must therefore be responsible for the death of *Ptdsr*-mutant mice. The frequently observed subcutaneous edema of various extents in *Ptdsr*-deficient homozygotes gave us a hint that *Ptdsr*-deficiency and lethality might be associated with cardiovascular problems. Indeed, very recently we have obtained strong evidence that *Ptdsr*-knockout mice die as a result of defects in heart development that are associated with specific cardiopulmonary malformations; (J.E. Schneider, J.B., S.D. Bamfort, A.D.G., C. Broadbent, K. Clarke, S. Neubauer, A.L. and S. Battacharya, unpublished observations).

In addition, we demonstrate that eye development requires a functional *Ptdsr *gene. *Ptdsr*-deficient embryos can be roughly divided into two categories. The first, severely affected group develops anophthalmia that correlates with formation of ectopic retinal-pigmented epithelium and induction of proliferation of underlying mesenchyme in the nasal cavity. This phenotype represents a completely novel lesion that to our knowledge has not been described before in any other mouse mutant. The second group shows normal external eye structures, although in this case retinal development is temporally delayed during mid-gestation, with persistent, abnormal morphogenesis of the inner granular retinal layer at later stages of embryogenesis. A possible explanation for these two phenotypes can be found in the expression pattern of the *Ptdsr *gene. Initially, *Ptdsr *is expressed throughout the whole developing nervous system, with exceptionally high levels in the anterior part of the forebrain. Later expression becomes more restricted to the developing retina and lens. Thus, *Ptdsr *might play an important role in early events of ocular morphogenesis, such as establishment and bisection of eye fields and formation of optic cups. These early eye-formation steps are closely interconnected with development of the forebrain [37,38] and the nose [39-41].

Interestingly, we occasionally observed serious malformations of forebrain and nasal structures in *Ptdsr*-knockout embryos that were associated with bilateral anophthalmia (see for example the mutant embryo in Figure 1g). This suggests that *Ptdsr *is involved in the regulation of differentiation processes within forebrain regions, and that ablation of *Ptdsr *function might secondarily affect early eye formation. Li *et al. *[31] found smaller lenses in *Ptdsr*-knockout mice and described the formation of retinal protrusions, although anophthalmia and specific differentiation defects of retinal cell layers were not reported in their study. Li *et al. *proposed [31] that the eye phenotype they observed could be explained by failed removal of apoptotic cells during eye development, but we think that the observed defects are unrelated to a failure of apoptotic cell clearance. A recent comprehensive kinetic analysis of apoptosis induction during mouse retinal development described four major peaks of apoptotic cell death [42]. This study demonstrated that there is an initial phase of cell death during the invagination of the optic cup (E10.5), followed by subsequent waves of apoptosis induction immediately before and after birth (E18.5 to postnatal day P2), and from postnatal days P9 to P10 and P14 to P16 [42]. Thus, besides the formation of the inner and outer layers of the optic cup in early eye development, other major phases of retinal cell apoptosis take place only postnatally and correspond to important periods in the establishment of neuronal connections. Furthermore, cell death during normal retinal development occurs in retinal layers distinct from the inner granular layer where we observed the most pronounced differentiation defects in the *Ptdsr *^-/- ^mutants described here. Other studies that connect the postnatal elimination of apoptotic photoreceptor cells to Ptdsr-mediated macrophage engulfment [43] should be interpreted with extreme caution as these studies were based on the monoclonal anti-Ptdsr antibody mAb 217G8E9 [26,43] (see below).

Consistent with the results of Li *et al. *[31], we found particular brain malformations in our *Ptdsr *^-/- ^mice. Exencephaly and hyperplastic brain phenotypes were observed at a low penetrance in *Ptdsr-*mutant mice (less then 4.5% of homozygotes), but these do not resemble to any extent the brain-overgrowth phenotypes of caspase- or *Apaf1*-knockout mice ([44], and references therein) in that we failed to identify any differences in the number or distribution of apoptotic cells or pyknotic cell clusters in the neuroepithelium of *Ptdsr *^-/- ^and *Ptdsr*^+/+ ^mice. Thus, reduced cell death or diminished clearance of apoptotic neural progenitor cells is unlikely to be the cause of the brain hyperplasia.

In summary, our studies demonstrate that *Ptdsr *is required for normal tissue differentiation, especially during the mid-gestation period when we observed the most severe differentiation delays in several organs of *Ptdsr*-knockout mice. The multiple defects in tissue differentiation cannot be explained by failure of apoptotic cell clearance, as this process is normal in our *Ptdsr*-knockout line. This result therefore indicates that *Ptdsr *has a novel, hitherto unexpected, role in promoting tissue maturation and terminal differentiation. Additional studies with conditionally targeted *Ptdsr*-deficient mice are required to investigate the role of spatial and temporal *Ptdsr *expression and function during tissue differentiation.

### *Ptdsr *is not essential for the clearance of apoptotic cells

Our studies demonstrate that Ptdsr is not a primary receptor for the uptake of apoptotic cells. Investigation of apoptotic cell clearance *in vivo *in *Ptdsr *^-/- ^embryos conclusively showed that removal of apoptotic cells is not compromised by ablation of *Ptdsr *function. Comparative analysis of ten different tissues and organs in *Ptdsr*^+/+ ^and *Ptdsr *^-/- ^animals at several stages of embryonic development and in neonates failed to identify impaired uptake of apoptotic cells at any time during development. Furthermore, phagocytosis assays *in vitro *demonstrated a completely normal uptake of apoptotic cells by *Ptdsr *^-/- ^macrophages, with some knockout macrophages showing loads even higher than wild-type of engulfed dead cells. These results are contrary to the expected role of Ptdsr in apoptotic cell clearance and to the reported findings of Li *et al. *[31] and Kunisaki *et al. *[32], as well as to a study done with a phosphatidylserine receptor null allele in *C. elegans *[45]. In previous studies in the mouse, the distribution and amount of apoptotic cells in *Ptdsr*-knockout and control animals were investigated in only a few tissues and at one [31] or two [32] developmental stages. Li *et al. *[31] examined lung, midbrain and retina at day E17.5 of gestation and identified apoptotic cells by TUNEL staining. Their findings must be interpreted with caution because remodeling of cellular structures by apoptosis in specific retina layers is known to occur mainly postnatally [42], and apoptosis plays an important physiological role in the maintenance and homeostasis of lung epithelium after birth or in pathological conditions involving pulmonary inflammation and not during lung development [46]. This postnatal role for apoptosis is in accordance with our data, as we rarely observed apoptotic cells in retina or lung tissue throughout embryogenesis in *Ptdsr*^+/+ ^and *Ptdsr *^-/- ^mice. Kunisaki *et al. *[32] analyzed TUNEL-stained sections of liver and thymus at days E13.5 and E16.5 of development in *Ptdsr*^+/- ^and *Ptdsr *^-/- ^embryos and found reduced rather than increased numbers of TUNEL-positive cells in *Ptdsr*-deficient embryos. Using co-localization of TUNEL-positive cells with F4/80-positive macrophages they suggested that *Ptdsr *^-/- ^embryos exhibited a three-fold increase in the frequency of unphagocytosed TUNEL-positive cells together with a severely reduced number of F4/80-positive cells. These results must be interpreted very carefully, however, as it is technically difficult to unambiguously identify engulfed target cells in individual macrophages in solid tissues by fluorescence microscopy.

In addition, our data suggest that during embryogenesis, macrophage-mediated clearance of apoptotic cells is not the only - or even the primary - mechanism for the removal of apoptotic cells. In many tissues where programmed cell death occurs as a prominent event during embryogenesis, such as remodeling of the genital ridge during gonad morphogenesis and differentiation of the neural tube, we found almost no co-localization of apoptotic cells and macrophages. This indicates that in these cases clearance of apoptotic cells is directly mediated by neighboring 'bystander' cells rather than by macrophages that have been recruited into areas where apoptosis occurs. Obviously these *in vivo *clearance mechanisms are not compromised by *Ptdsr*-deficiency in our knockout mutant. This finding is in line with studies in macrophageless *Sfpi1*-knockout embryos that are deficient for the hematopoietic-lineage-specific transcription factor PU.1. Here, the phagocytosis of apoptotic cells during embryogenesis is taken over by 'stand-in' mesenchymal neighbors [47]. As recognition of phosphatidylserine is thought to be a universal engulfment mechanism for all cells that are able to phagocytose apoptotic cells, it is very striking that apoptotic cell clearance mediated by non-professional bystander cells is also not compromised by *Ptdsr*-deficiency.

In contrast to Li *et al. *[31], we did not observe any impairment in the uptake of apoptotic cells by *Ptdsr *^-/- ^macrophages *in vitro*. We performed phagocytosis assays *in vitro *with fetal-liver-derived macrophages, while in their assays, Li and colleagues used thioglycollate-elicited peritoneal macrophages after adoptive transfer of *Ptdsr *^-/- ^hematopoietic stem cells. The different results obtained in the two studies are puzzling; they might be due to the use of different macrophage or cell populations. We and Kunisaki *et al. *[32] found that *Ptdsr*-deficiency is to some extent associated with defects in hematopoiesis. Thus, it seems possible that recruitment and activation/differentiation of macrophages after adoptive transfer and thioglycollate elicitation are affected by *Ptdsr*-deficiency. We do not think that the different results observed in *Ptdsr*-knockout mice in a mixed C57BL/6 × 129 background and in a pure C57BL/6J background can be attributed to genetic background effects: comparison of apoptotic cell engulfment efficacies of thioglycollate-elicited macrophages from 129P2/OlaHsd and C57BL/6J mice did not show any differences in apoptotic cell uptake (J.B. and A.L., unpublished observations). Moreover, in contrast to our studies, neither Li *et al. *[31] nor Kunisaki *et al. *[32] determined phagocytotic engulfment indexes for *Ptdsr*-deficient macrophages.

Interestingly, we observed differences between *Ptdsr*^+/+ ^and *Ptdsr *^-/- ^macrophages in the secretion of pro- and anti-inflammatory cytokines after stimulation with LPS and apoptotic cells. This provides evidence that cellular activation and effector mechanisms are impaired in *Ptdsr*-deleted macrophages. It remains to be determined which classical pathways of macrophage activation and function involve *Ptdsr*. This is especially important in light of recent findings that demonstrated nuclear localization of the Ptdsr protein [29].

Most strikingly, the recently published data regarding the genetic ablation or perturbation of phosphatidylserine receptor function in *C. elegans *are also contradictory. Wang *et al. *[45] reported that *psr-1*, the *C. elegans *homolog of *Ptdsr*, is important for cell-corpse engulfment, whereas *psr-1 *RNAi studies performed by Arur *et al. *[25] yielded, in this respect, no phenotype. Moreover, Wang and colleagues hypothesized on the basis of their data that *psr-1 *might act to transduce an engulfment signal upstream of Ced-2 (Crk II), Ced-5 (Dock 180), Ced-10 (Rac 1) and Ced-12 (Elmo) in one of the two cell-corpse engulfment pathways in the worm [45]. But the loss-of-function phenotype of *psr-1 *mutants and the complementation phenotypes in overexpressing transgenic worms shown by Wang *et al. *[45] are rather weak as compared to the classical *C. elegans *engulfment mutants [8].

Many previous functional studies that reported a requirement for Ptdsr for the phagocytosis of apoptotic cells used the monoclonal anti-Ptdsr antibody mAb 217G8E9 [26]. This antibody was used in Ptdsr binding and blocking experiments, as well as in subcellular localization studies, which led to the conclusion that Ptdsr is a transmembrane receptor critical for signal transduction at the engulfment interface. More recently it was used in binding assays to show that the human and worm Ptdsr molecules can recognize phosphatidylserine [45]. In the course of the study presented here, we stained immunohistochemically for Ptdsr with mAb 217G8E9 on wild-type and *Ptdsr*-deficient macrophages and fibroblasts (see Additional data file 1, Figure S2 and data not shown). To our surprise, we observed similar staining patterns with cells of both genotypes. Furthermore, using a Ptdsr-peptide array we found that mAb 217G8E9 can bind weakly to a Ptdsr peptide, explaining the original isolation of Ptdsr cDNA clones by phage display [26]; but the antibody mainly recognizes additional, as-yet unknown, membrane-associated protein(s) (see Additional data file 1, Figure S2). Experiments that have used this antibody should therefore be interpreted with great caution as they might come to be viewed in a different light.

## Conclusion

Our results demonstrate that *Ptdsr *is essential for the differentiation and maturation of multiple tissues during embryogenesis. Ablation of *Ptdsr *function results in neonatal lethality and severe defects in the morphogenesis of several organs. The developmental malformations cannot be explained by impaired clearance of apoptotic cells, a process that proved to be normal in *Ptdsr*-deficient mice. This opens up the possibility either that there is an as-yet unknown Ptdsr receptor, which might act as a primary phosphatidylserine recognition receptor, or that recognition of phosphatidylserine and subsequent apoptotic cell engulfment and anti-inflammatory signaling are mainly mediated through phosphatidylserine bridging proteins and their cognate receptors. Although *Ptdsr *^-/- ^macrophages were not impaired in their ability to phagocytose apoptotic cells, they showed reduced cytokine responses after stimulation. Further work will be required to determine the molecular mechanisms of these newly recognized *Ptdsr *functions during development.

## Materials and methods

### Construction of the targeting vector and generation of *Ptdsr*-knockout and gene-trap mice

#### Targeting vector

A *Ptdsr*-containing bacterial artificial chromosome (BAC) clone (GenBank accession number AC091694; RP-23-316F3) was isolated by sequence homology from a C57BL/6J genomic BAC library (RP-23; BACPAC Resources, Oakland, USA). A 14.5 kb *Kpn*I/*Bam*HI fragment containing the entire *Ptdsr *locus and 5' and 3' flanking regions was subcloned from this BAC clone and a 1.9 kb *Rsr*II/*Aat*II fragment containing exons I and II of the *Ptdsr *gene was replaced by a 1.2 kb *lox*P-flanked neomycin-resistance gene cassette (*neo*).

#### Homologous recombination in ES cells and generation of germ-line chimeras

Bruce4 ES cells were transfected with *Kpn*I-linearized targeting vector and selected with G418. ES-cell clones resistant to G418 were isolated and analyzed by Southern blot analysis for homologous recombination events within the *Ptdsr *locus. Chimeric mice were produced by microinjection of two independent homologous recombinant (*Ptdsr*^+/-^) ES cells into BALB/c blastocysts and transfer to pseudopregnant foster mothers followed by development to term. Chimeric males were mated with C57BL/6J females. From the two selected ES-cell clones, one successfully contributed to the germ-line. Germ-line transmission of the mutant allele was verified by PCR and Southern blot of genomic DNA from black coat-color F1 offspring.

#### Ptdsr gene-trap and generation of germ-line chimeras

An ES-cell line carrying a *β-geo *gene-trap vector in the *Ptdsr *locus was identified by searching the BayGenomics database (BayGenomics, San Francisco, USA; [48]) with the full-length *Ptdsr *cDNA. A single ES-cell line was identified carrying the gene-trap in intron V, between exons V and VI of the *Ptdsr *gene. Chimeric mice were generated by microinjection into CB20 blastocysts and transfer to pseudopregnant foster mothers. Chimeric males were mated with 129P2/OlaHsd females. Germ-line transmission of the mutant gene-trap allele was verified by expression analysis using *β*-galactosidase staining and RT-PCR.

### Genotype analysis

The genotypes of embryos or animals were determined by PCR analysis and confirmed by Southern blot. Genomic DNA for PCR was prepared from extraembryonic membranes or tail clips using a non-organic tail-DNA extraction protocol [49]. High molecular weight genomic DNA for Southern blotting was prepared according to standard protocols. For PCR analysis the wild-type *Ptdsr *allele was detected using forward primer 1 (5'-GACACTGTCCATGGCAAACAC-3') and reverse primer 2 (5'-TAAAGTCGCCTTCCAGAAGATT-3'). The primer 1 site is located 5' to the deletion and the primer 2 site within the deletion. This primer pair amplified a fragment of approximately 300 bp from wild-type and *Ptdsr*^+/- ^mice but not from *Ptdsr *^-/-^mutants. To detect the mutant *Ptdsr *allele, genomic DNA was also amplified using primer 1 and reverse primer 3 (5'-CCACACGCGTCACCTTAATA-3'), which corresponds to a sequence in the *neo *cassette. In this case, a 500 bp fragment was detected in mice heterozygous or homozygous for the mutant allele, while no signal was detected in wild-type mice. For Southern blot analysis, genomic DNA (30 μg) was digested overnight with *Bam*HI (30 U; Roche Diagnostics GmbH, Mannheim, Germany) and *Sca*I (30 U; Roche), fractionated on a 0.8 % agarose gel, transferred to a nylon membrane (Hybond N; Amersham Biosciences Europe GmbH, Freiburg, Germany) and hybridized with 5' and 3' flanking probes. The *Bam*HI digest was hybridized with a *Ptdsr*-specific 5' flanking probe, and Southern blot gave a single 17.2 kb band for wild-type (^+/+^), an 11.6 kb band for homozygous (^-/-^) and both bands for heterozygous (^+/-^) mice. The *Sca*I digest was hybridized using a 3' flanking probe, and Southern blot gave a single 12.4 kb band for wild-type, a 17.2 kb band for homozygous and both bands for heterozygous mice.

### Northern blot analysis

Total RNA was isolated from homogenized embryos using TRIZOL reagent (Invitrogen GmbH, Karlsruhe, Germany). For northern blots, either total RNA (30 μg) was extracted from embryos, electrophoresed and transferred to a nylon membrane (Hybond N; Amersham) or a polyA^+ ^RNA northern blot (OriGene Technologies Inc., Rockville, USA) was hybridized using as the probe a *Ptdsr *fragment amplified from wild-type cDNA using forward primer 5'-GTTCCAGCTCGTCAGACTCG-3' and reverse primer 5'-TGCCCCTAAGACATGACCAC-3'. In all experiments the same membrane was re-hybridized with a *β-actin *probe (OriGene) to confirm that equivalent RNA levels were present in each lane. Northern blotting indicated that homozygous mutant embryos did not express *Ptdsr *mRNA and heterozygous mutant embryos expressed only reduced amounts of *Ptdsr *mRNA.

### Western blot analysis

Embryos (E13.5) for protein isolation were homogenized in lysis buffer containing 1 × PBS, 1% Nonidet P-40, 0.5% sodium deoxycholate, 0.1% SDS and protease inhibitor cocktail (CompleteMini; Roche). Equal amounts (25 μg) of protein lysate were separated by SDS-polyacrylamide gel electrophoresis and transferred onto a PVDF membrane (Millipore, Billerica, USA) according to standard protocols. Western blots were done using a specific antibody to Ptdsr (PSR N-20, sc-11632; Santa Cruz Biotechnology Inc., Santa Cruz, USA) and β-actin (ab-6276; Abcam, Cambridge, UK) as described by the supplier. Secondary antibodies conjugated to horseradish peroxidase were from Santa Cruz and Abcam, used as described by the supplier, and detection was performed with an enhanced chemiluminescence system (ECLPlus; Amersham).

### Animal experiments

Wild-type C57BL/6J and 129P2/OlaHsd mice were obtained from Jackson Laboratories (Bar Harbor, USA) and Harlan UK (Bicester, UK), respectively. All mice were housed in individually ventilated cages in a specific pathogen-free environment with a 12 h light-dark cycle and were fed a regular unrestricted diet. The GBF's routine surveillance program screened for selected pathogens. The *Ptdsr*^*tm1Gbf *^mutant was crossed to C57BL/6J mice to establish the co-isogenic C57BL/6J-*Ptdsr*^*tm1Gbf*^ mouse line. All studies were approved by the appropriate authorities.

### Isolation of embryos

Heterozygous male and female mice were intercrossed in order to obtain *Ptdsr*-deficient progeny. Females were daily monitored for vaginal plugs, and noon of the day of plug detection was defined as E0.5. Embryos at indicated time points were dissected in sterile PBS, washed in ice-cold PBS and transferred to cold fixative. Extra-embryonic membranes were kept and used for genotyping. *Ptdsr *^-/- ^embryos and their wild-type littermates were used for experiments.

### Histology, TUNEL staining and immunohistochemistry

Embryos for histology and immunohistochemistry were harvested and fixed in 10% neutral-buffered formalin, dehydrated through a graded series of alcohol, embedded in paraffin, sagittally sectioned at 5 μm intervals, and every fifth section was processed for hematoxylin and eosin (H&E) staining according to standard protocols. Remaining sections of wild-type and *Ptdsr *^-/- ^specimens were used for immunohistochemistry. For detection of apoptotic cells and macrophages, anti-aCasp3 (an antibody specific for activated caspase 3; R&D Systems, Minneapolis, USA) and anti-F4/80 (Serotec GmBH, Düsseldorf, Germany; #MCA 1957) antibodies were used as described by the supplier. Detection was performed using indirect streptavidin with biotinylated secondary antibodies and cobalt-enhanced diaminobenzidine (brown) or fast-red (red) as chromogens. Sections were counterstained with hematoxylin. For whole-mount terminal deoxynucleotidyl transferase-mediated UTP end labeling (TUNEL), limb buds were dissected from E12.5 and E13.5 embryos, fixed in 4% paraformaldehyde and processed for analysis as previously described [50].

### Preparation of fetal liver-derived macrophages (FLDMs)

Fetal livers were excised from embryos at E12.5 and E13.5, respectively, washed in PBS and dissociated enzymatically for 60 min at 37°C. The digestion buffer (150 μl per liver) comprised 0.6 U/ml dispase I (Roche), 0.1% collagenase D (Roche), 10 U DNase (Roche), and 20% FCS in PBS. X-Vivo 15 medium (Cambrex, East Rutherford, USA) was added to the resulting cell suspension, and after centrifugation (200 × *g*; 3 min) cells were resuspended in X-Vivo 15 medium supplemented with 50 ng/ml macrophage colony-stimulating factor (M-CSF; Sigma-Aldrich, St. Louis, USA) and cultured on non-treated tissue-culture dishes at 37°C with 5% CO_2_. Every second or third day the medium was changed by centrifugation. Following withdrawal of M-CSF on day 6 after excision, adherent cells were cultured for an additional 24-48 h in X-Vivo 15 medium.

### Macrophage phagocytosis assays

For preparation of monolayer cultures of macrophages, FLDMs were plated on glass coverslips in 24 well plates (2 × 10^5 ^cells per well) in X-Vivo 15 medium. For preparation of apoptotic target cells, primary thymocytes were harvested from the thymus of 4- to 8-week-old C57BL/6J mice, stained with TAMRA for 15 min, and apoptosis was induced either by treating cells with 5 μM staurosporine in medium for 4 h at 37°C or by culturing cells in medium overnight. The efficacy of apoptosis induction was compared in thymic target cells and controls by FACS analysis. On average, 60% of the cells of the resulting population were apoptotic, with exposed PS on their surface, and less than 5% of the cells were necrotic, as confirmed by FITC-annexin V and propidium iodide staining. The apoptotic thymocytes obtained were washed with PBS and added to the prepared FLDM cultures (ratio 10:1). Phagocytosis was then allowed to proceed at 37°C and 5% CO_2_. After the indicated time periods, the uptake of apoptotic cells by FLDMs was stopped by intensive washing of co-cultures with cold PBS to remove unphagocytosed cells. To measure phagocytosis of apoptotic thymocytes, macrophages were further processed for immunofluorescence analysis. Cells were fixed in 4% paraformaldehyde, blocked in 0.5% BSA/PBS and stained with an anti-F4/80 antibody (Serotec) followed by a secondary antibody coupled to Alexa 488 (Molecular Probes Inc., Eugene, USA). Coverslips were mounted on slides and engulfed thymocytes were enumerated by fluorescence microscopy. The percentage of phagocytosis was calculated by counting at least 300 macrophages and determining the number of macrophages that had engulfed apoptotic thymocytes. The phagocytotic index was calculated according to the following formula: phagocytotic index = (total number of engulfed cells/total number of counted macrophages) × (number of macrophages containing engulfed cells/total number of counted macrophages) × 100. The experiments were performed at least three times, each time in triplicate, and the counting was done by three different investigators.

### Measurement of macrophage cytokine production

Monolayer cultures of FLDMs and apoptotic thymocytes were prepared as described above. FLDMs were incubated with medium, LPS (10 ng/ml), apoptotic cells (ratio 1:10) or both for the determination of IL-10, TGF-β1 or TNF-α levels after co-culture for 22 h. For TNF-α quantification at various time points, FLDMs were cultured with a high concentration of LPS (100 ng/ml). Culture supernatants were harvested and TNF-α (Mouse TNF-α OptEIA set; BD Biosciences, Heidelberg, Germany) and TGF-β1 (Quantikine, TGF-β1 immunoassay; R&D Systems) were measured by ELISA as described by the supplier. IL-10 in culture supernatants was determined by a cytometric bead assay (Mouse inflammation CBA; BD Biosciences) as indicated in the manual. Data are presented as mean ± SEM from at least three independent experiments, each carried out in triplicate. Analysis of the results used the Wilcoxon-signed rank test; *p *values below 0.05 were considered significant.

## Additional data files

Additional data file 1 contains: Figure S1 showing the localization of apoptotic cells and macrophages in the subcutis of developing embryos; and Figure S2 showing immunohistochemical staining of the Ptdsr protein in macrophages derived from wild-type and *Ptdsr*-knockout mice.

## Supplementary Material

Additional data file 1 Figure S1 showing the localization of apoptotic cells and macrophages in the subcutis of developing embryos; and Figure S2 showing immunohistochemical staining of the Ptdsr protein in macrophages derived from wild-type and *Ptdsr*-knockout mice Click here for additional data file

## Figures and Tables

**Table 1:** Penetrance of phenotypes in *Ptdsr *^-/- ^mice from E9.5 to E18.5, as detected by gross morphology

Dysmorphic phenotypes	Ratio in analyzed mice (affected/total)	Penetrance (%)
Head malformations	9/198	4.5
cleft	4/198	2.0
others	5/198	2.5
Edema (E12.5-E15.5)	15/155	9.7
Pale appearance (= E14.5)	72/72	100
Ocular lesions	28/198	14.1
unilaterally absent eyes	21/198	10.6
right	16/198	8.1
left	5/198	2.5
bilaterally absent eyes	7/198	3.5

Subsets of the major categories of malformation are indicated by indentation.
