# Supplementary material for: The phosphatidylserine receptor has essential functions during embryogenesis but not in apoptotic cell removal
Source: J Biol. 2004 Aug 23;3(4):15. doi: 10.1186/jbiol10 (PMC549712; doi:10.1186/jbiol10)
Supplement: Additional data file 1 — Figure S1 showing the localization of apoptotic cells and macrophages in the subcutis of developing embryos; and Figure S2 showing immunohistochemical staining of the Ptdsr protein in macrophages derived from wild-type and Ptdsr-knockout mice [file jbiol10-s1.pdf]

Additional Figures

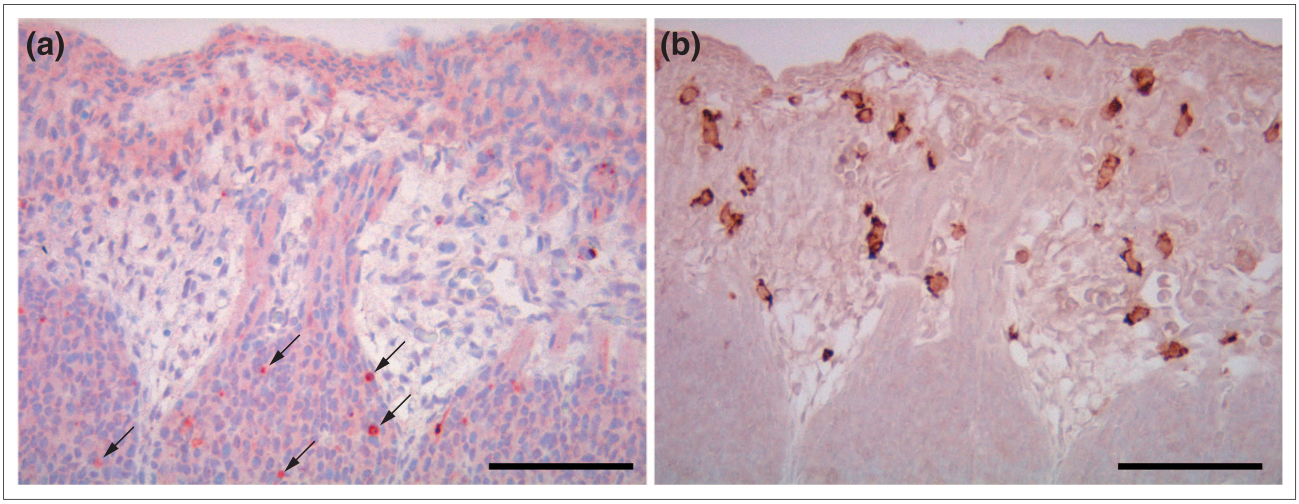

Figure S1
Additional evidence that apoptotic cells do not co-localize with F4/80-stained macrophages during mouse development: localization of apoptotic cells and macrophages in the subcutis of developing embryos (E16.5). Examination of serial sagittal sections stained for (a) aCasp3 (red, apoptotic cells) or (b) F4/80 (brown, macrophages) reveals that apoptotic cells and macrophages are not co-localized. The same observation was made in Ptdsr$^{-/-}$ embryos and in all organs and developmental stages analyzed in this study. Scale bar, 100 μm.

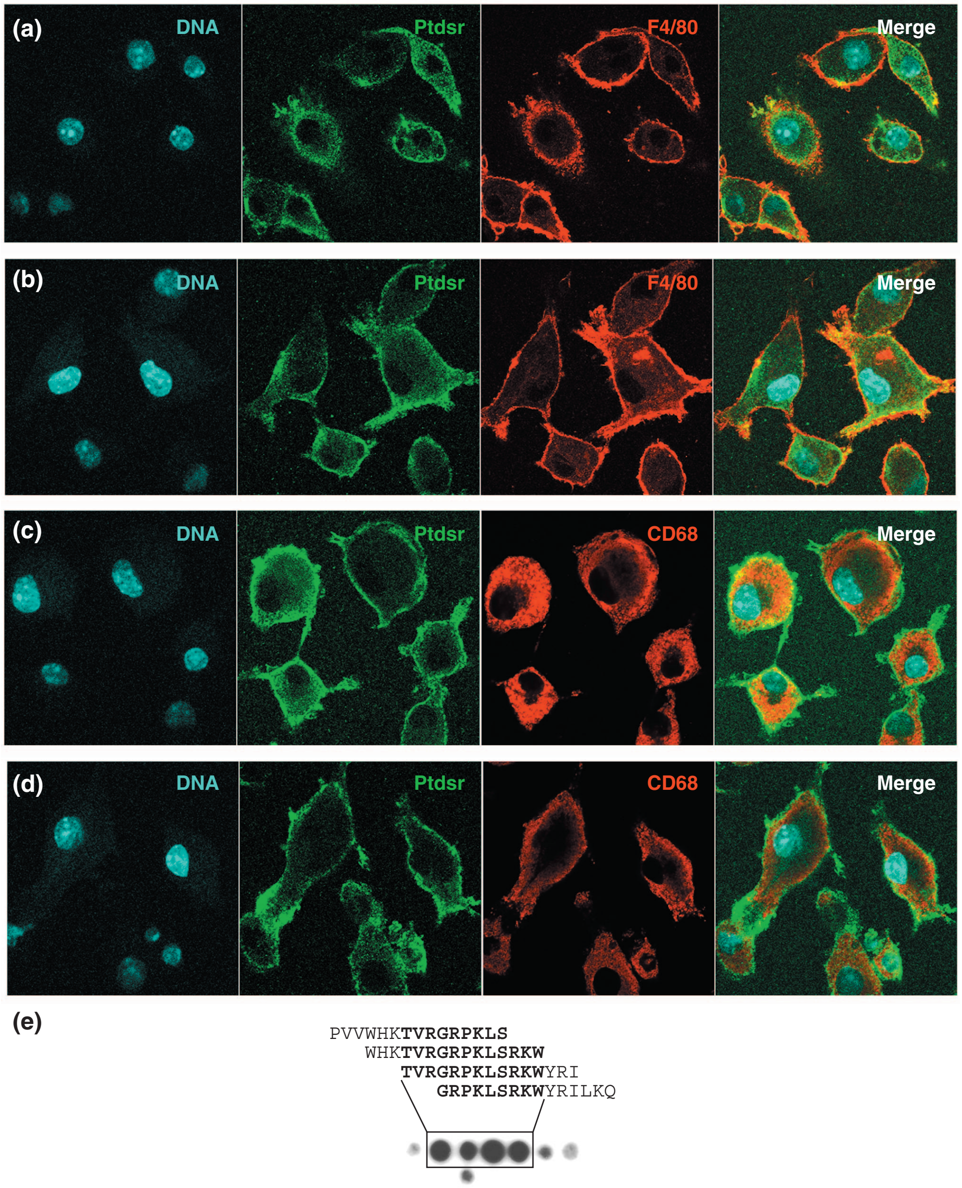

Figure S2

Immunohistochemical staining of Ptdsr$^{+/+}$ and Ptdsr$^{-/-}$ macrophages with the anti-Ptdsr antibody mAb 217G8E9. (a,c) Wild-type and (b,d) knockout fetal-liver-derived macrophages (FLDMs) were stained with the mouse monoclonal IgM anti-Ptdsr antibody mAb 217 [26] (Cascade BioScience, Winchester, USA; #ABM-3099), and the localization of the mAB 217 antigen was analyzed in (a,b) non-permeabilized and (c,d) permeabilized cells. In both genotypes ($^{+/+}$ and $^{-/-}$) the staining was indistinguishable (compare a with b or c with d) and did not change under different experimental conditions. The permeabilized FLDMs (c,d) were counter-stained with an antibody against the lysozomal glycoprotein CD68 (Serotec, #MCA 1957) and the non-permeabilized macrophages (a,b) with an antibody against the macrophage cell-surface glycoprotein F4/80 (Serotec, #MCAP 497). DNA was stained in all cells with Hoechst 33342 (Molecular Probes) to visualize nuclei. As secondary antibodies, Alexa488-anti-mouse IgM and Alexa568-anti rat IgG antibody (Molecular Probes) were used, respectively. Using fibroblasts or FLDMs pretreated with β-glucan for 24 h showed no difference in staining between the two genotypes. Only a slight increase in the expression of the antigen recognized by the antibody mAB 217 could be detected after pretreatment of macrophages with β-glucan (data not shown). (e) Recognition of linear epitopes of Ptdsr protein. A peptide array representing the carboxy-terminal part (amino acids 289-403) of the Ptdsr protein was chemically synthesized on cellulose sheets and incubated with the monoclonal antibody mAB 217 [52]. Peptides mainly recognized by the antibody are boxed; the deduced amino-acid sequences of the epitopes recognized are in bold.
